# Comment on Scaglioni et al. Present and Future of Autologous Breast Reconstruction: Advancing Techniques to Minimize Morbidity and Complications, Enhancing Quality of Life and Patient Satisfaction. *J. Clin. Med.* 2025, *14*, 2599

**DOI:** 10.3390/jcm15020590

**Published:** 2026-01-12

**Authors:** Franck Dupuy, Fabien Boucher, Ali Mojallal, Guillaume Henry

**Affiliations:** Plastic and Reconstructive Surgery, Croix Rousse Hospital, 69004 Lyon, France; fabien.boucher@chu-lyon.fr (F.B.); dr.mojallal@gmail.com (A.M.); guillaumehen@orange.fr (G.H.)

We read with great interest the article by Scaglioni et al. [1] and fully agree with their insightful discussion on recent innovations in autologous breast reconstruction. The authors provide a comprehensive review of emerging techniques, including perforator-to-perforator anastomosis, rib-sparing vessel preparation, superficial-system-based flaps such as SIEA/SIEAP and SCIP, as well as the promising emergence of robotic assistance in microsurgery. These developments represent meaningful steps toward reducing surgical morbidity and refining reconstructive outcomes.

However, our own experience suggests that innovation in autologous breast reconstruction extends beyond technical refinement alone. Substantial progress arises from the early integration of the reconstructive surgeon—or ideally a dual-trained breast surgeon—into oncologic planning. Early reconstructive input shapes mastectomy design, lymph node strategy, and aesthetic anticipation, while ensuring alignment with patient expectations. When this early involvement is combined with efficient perioperative organization, a natural consequence emerges: the democratization of immediate breast reconstruction. Many of the traditional barriers to immediate autologous reconstruction are organizational rather than technical, and early reconstructive participation directly addresses these limitations, as highlighted in recent analyses underscoring the central role of plastic surgeons in breast cancer care [2].

From this approach naturally follows the broader optimization of the reconstructive pathway, aiming to reduce the psychological and physical burden of treatment and streamline the overall process. A key component of this strategy is minimizing both the number and the impact of surgical procedures. In our practice, we prioritize limiting operative stages, achieving immediate symmetry through simultaneous contralateral reduction or mastopexy [3], positioning the abdominal donor-site scar optimally from the outset, and shaping the DIEP flap as close as possible to the intended final result at the index procedure.

To support this strategy, our practice is embedded within a structured Enhanced Recovery After Surgery (ERAS) pathway, consistent with the ERAS Society recommendations for breast reconstruction [4]. This includes comprehensive preoperative counseling, streamlined imaging—frequently using remote or pre-existing CT scans to avoid unnecessary examinations [5]—day-of-surgery admission, and strict intraoperative efficiency.

Several operative principles have significantly improved outcomes in our center. These include standardized room organization and double-team operating, simultaneous flap harvest and mastectomy, early selection of a single dominant perforator, venous coupler-assisted anastomoses [6], exclusive use of surgical loupes, proven to be as safe as the operating microscope [7], and systematic de-epithelialization of the flap, allowing efficient shaping and precise assessment of dermal bleeding before and after anastomosis.

Importantly, we rely primarily on clinical perfusion indicators—especially the reproducibility and quality of dermal bleeding—rather than on indocyanine green (ICG) angiography. In our experience, routine ICG use prolongs operative time, disrupts workflow, and increases technical complexity without improving outcomes, provided clinical assessment is rigorous. This aligns with strong evidence identifying operative time as a key predictor of postoperative morbidity in DIEP reconstruction [8,9].

On the anesthetic side, a morphine-sparing regimen combined with targeted loco-regional blocks facilitates immediate mobilization upon return to the ward. Typical discharge occurs between postoperative days 2 and 4, consistent with recent work supporting early discharge in optimized settings [10].

Regarding postoperative monitoring, our experience confirms the safety of burying the flap without a skin paddle in both immediate and delayed settings, provided intraoperative perfusion assessment is conclusive. This aligns with recent studies demonstrating the reliability of buried flaps in appropriately selected patients [11,12,13].

These organizational and technical refinements have significantly reduced postoperative complications, including donor-site morbidity—with near absence of abdominal bulging—and the need for secondary procedures, while enabling more consistent symmetry at the first stage, in agreement with data highlighting the long-term benefits of workflow optimization [9,10,11,12,13,14].

Finally, we believe that the future of autologous breast reconstruction also relies on the effective transmission of these techniques to younger surgeons. Within experienced teams, this occurs through true surgical companionship, following a progressive model in which trainees first observe, then operate under direct supervision, and ultimately perform the procedure autonomously with senior support. As illustrated by Fischer et al. [15], such structured mentorship significantly shortens the learning curve while maintaining high standards of safety and efficiency.

Innovation, therefore, does not rely solely on technological sophistication. It lies equally in demystifying the procedure, optimizing team efficiency, broadening access to immediate reconstruction, and ensuring safe, reproducible surgical transmission across generations.
